# A case in which a near-infrared ray catheter (NIRC) was effectively applied in laparoscopic sigmoidectomy for a sigmoidovesical fistula

**DOI:** 10.1016/j.ijscr.2021.106641

**Published:** 2021-12-02

**Authors:** Atsuko Okamoto, Shunjin Ryu, Takahiro Kitagawa, Rui Marukuchi, Keigo Hara, Ryusuke Ito, Yukio Nakabayashi

**Affiliations:** Department of Digestive Surgery, Kawaguchi Municipal Medical Center, Japan

**Keywords:** Colovesical fistula, Fluorescence, Ureteral catheter

## Abstract

A 40-year-old man visited the hospital for a refractory urinary tract infection. A sigmoidovesical fistula resulting from a sigmoidovesical diverticulum was diagnosed, and laparoscopic surgery was performed. To avoid ureteral injury during surgery for highly advanced cancer and inflammatory diseases, a ureteral stent is generally placed before the procedure. However, in this case, surgery was performed using a near-infrared ray catheter (NIRC), which emits fluorescence when irradiated with near-infrared light. By clearly observing the pathway of the ureter via near-infrared light, the integrity of the ureter could be preserved, and sigmoidectomy was safely performed. The visual navigation of the ureter with NIRC was especially useful during surgery for a colovesical fistula with marked surrounding inflammatory changes and a high risk of ureteral damage.

## Highlights

• To avoid ureteral injury during surgery, surgery was performed using a near-infrared ray catheter (NIRC)
• NIRC could be observed clearly the pathway of the ureter via near-infrared light.
• The visual navigation of the ureter with NIRC was especially useful during surgery for a colovesical fistula.

## 1 Introduction and importance

The most common disorder due to a colovesical fistula is diverticular disease of the colon [1]. Due to the aging of society, cases of diverticular disease of the colon have been increasing in recent years. An acute abdominal condition is observed in 10– 25% of cases of diverticula in the colon, and approximately 30% of patients with acute inflammation have been reported to develop complications, such as perforation, abscess formation, and fistulation [2]. Previously, open sigmoidectomy was the standard treatment for sigmoidovesical fistulas caused by diverticula of the colon. In recent years, however, the usefulness of laparoscopic surgery has been reported [2], [3]. A ureteral stent is usually placed before surgery to avoid ureteral injury during surgery for inflammatory diseases. However, laparoscopic surgery does not allow manual palpation of the ureteral stent, and no evidence of ureteral injury prevention has been reported [4].

In this case, a fluorescence-emitting near-infrared ray catheter (NIRC)® (Nippon Covidien) that emits fluorescence when irradiated with near-infrared light was placed in the ureter before surgery. Then, laparoscopic sigmoidectomy could be safely performed by visualizing the pathway of the ureter with the fluorescence produced by near-infrared light.

To date, one report of sigmoidovesical fistula surgery using NIRC have been published. We report this method here because it could be considered especially useful.

## 2 Case presentation

### 2.1 Patient

A 40-year-old man.

### 2.2 Main complaints

Fever and pneumaturia.

### 2.3 Medical history

The patient consulted his previous doctor for fever and pneumaturia. He was diagnosed with a urinary tract infection and treated with antibiotics, but his condition did not improve, and he was referred to our hospital for a suspected refractory urinary tract infection. A sigmoidovesical fistula was diagnosed after careful examination, and surgery was subsequently performed.

### 2.4 Past history

No notable history.

### 2.5 Present condition

Height, 178.0 cm; weight, 60.5 kg. No obvious abnormalities were observed on abdominal examination.

### 2.6 Blood test findings at the initial medical examination

White blood cells (WBCs), 4300/μL; C-reactive protein (CRP), 0.04 mg/dL; carcinoembryonic antigen (CEA), 3.4 ng/mL; and carbohydrate antigen 19-9 (CA19-9), 17.8 U/L. No inflammatory response or increases in tumor markers were observed.

### 2.7 Plain abdominal computed tomography (CT) scan findings

Air was observed in the bladder, and multiple diverticula were found in the adjacent sigmoid colon. In addition, the sigmoid colon and bladder had cord-like lesions with contrast-enhanced margins, and the patient was diagnosed with a sigmoidovesical fistula.

### 2.8 Lower endoscopy

On colonoscopy, many diverticula were observed in the sigmoid colon, and a bulge due to extramural pressure 30 cm from the anal verge (AV) was observed. Pus discharge from the same site was noted, and biopsy showed a Group 1 infection.

### 2.9 Cystoscopy

The bladder trigone was edematous, and a fistula centered between the bilateral ureteral ostia was identified.

### 2.10 Operative findings

**Fig. 1:**
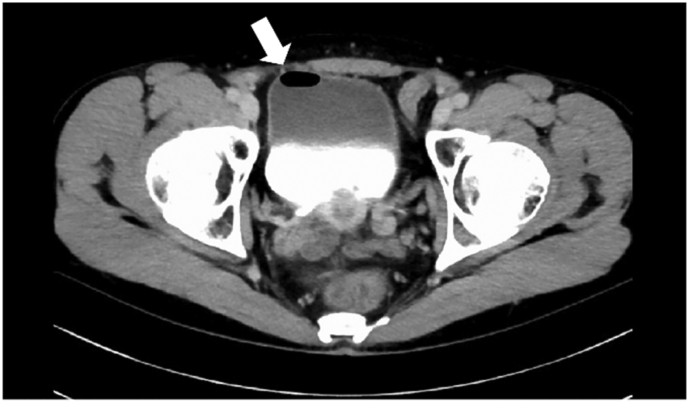
The white arrow shows the fistula between the sigmoid colon and bladder.

**Fig. 2:**
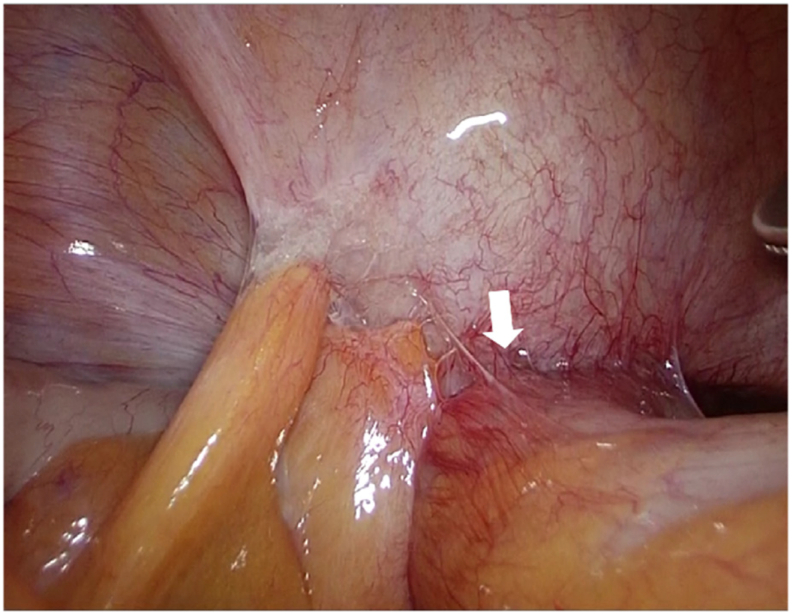
The open white arrows show the ureter (NIRC). The white arrows show the fistula between the sigmoid colon and bladder.

**Fig. 3:**
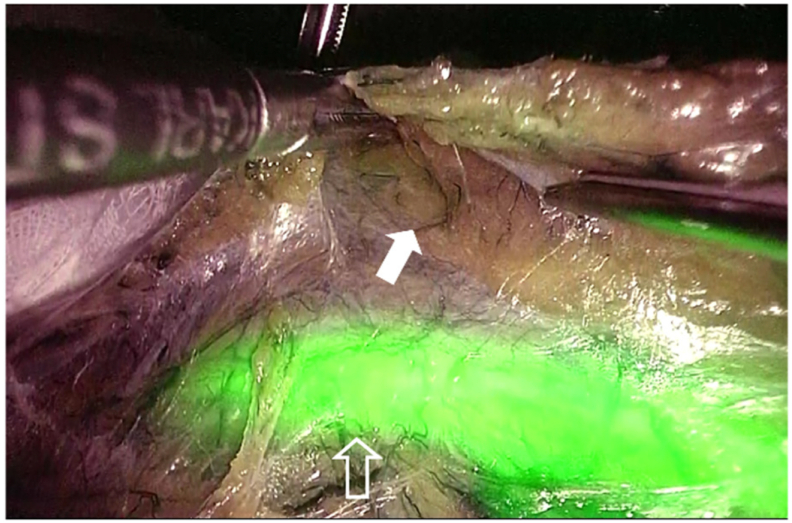
The open white arrow shows the ureter (NIRC). The white arrow shows the sigmoid mesentery. NIRC was useful for the mobilization of the sigmoid mesentery.

**Fig. 4:**
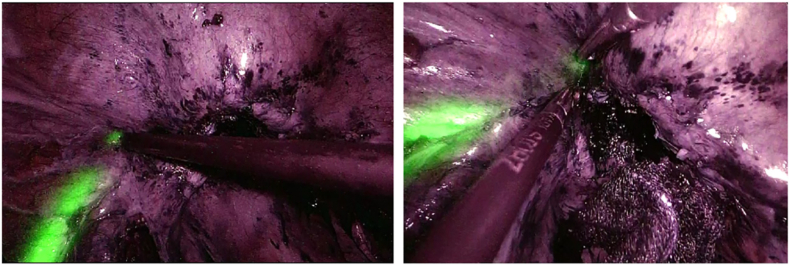
The ureteral orifice was confirmed by pressing the bladder wall with forceps.

Under general anesthesia, an NIRC® (Nippon Covidien) was placed in both ureters using a cystoscope with the patient in the lithotomy position. Endoscopic surgery with five ports was performed, and the abdominal cavity was visualized. The anterior surface of the sigmoid colon and the bladder trigone were rigidly adhered, and the sigmoid colon exhibited edema due to increasing inflammation (Fig. 1). To avoid ureteral injury, the sigmoid colon was mobilized using a medial approach guided by the fluorescence of the NIRC (Fig. 2). The fistula was dissected on the colon side, and the ureter pathway was confirmed during dissection using the NIRC (Fig. 3). In this case, the lesion was centered between the bilateral ureteral ostia, and the risk of ureteral injury was high. However, ureteral injury was prevented by performing surgery while clearly observing the pathway of the ureter using the NIRC. (Fig. 4). Indigo carmine and approximately 200 mL of saline were infused through a urethral balloon, but no obvious leakage into the abdominal cavity was observed. The bladder tissue in the fistula was closed with four stiches on the serosal side. After the sigmoid colon was dissected and the specimen was removed, anastomosis was performed using the double stapling technique to complete the surgery. The operative time was 281 min, and the blood loss was 5 mL.

### 2.11 Postoperative course

The patient's postoperative course was good, and he was discharged 10 days after the surgery without complications. The urethral balloon was removed in the outpatient department 13 days after the surgery.

### 2.12 Pathology

Granulation tissue in contact with the diverticula was found on the serosal side of the sigmoid colon. No malignant findings were identified, and the fistula was determined to be caused by diverticulitis.

## 3 Discussion

Previously, depending on the case, open sigmoidectomy and partial resection of the bladder were the standard treatments for sigmoidovesical fistulas caused by diverticula in the colon. In recent years, however, the usefulness of laparoscopic surgery has also been reported [2], [3].

The magnifying effect of laparoscopy has been suggested to facilitate the prevention of urinary tract injury and minimize complications by obviating unnecessary cystectomy. However, in cases in which a bladder trigone fistula or severe inflammation is expected, an artificial anus is created to avoid urinary tract injury. Two-stage colectomy and simultaneous or three-stage artificial anus closure should be performed after reduction of the fistula [5]. Two or three stages of surgery may be required, reflecting the high degree of invasiveness of this treatment for a benign disease. In addition, urinary diversion is required in some cases, and laparoscopic and endoscopic cooperative surgery using a cystoscope has been reported to minimize the extent of bladder resection and preserve the ureteral opening [6].

In recent years, fluorescence navigation in laparoscopic surgery has attracted attention. Indocyanine green (ICG) emits fluorescence when irradiated with near-infrared light, enabling the visualization of in vivo information, such as blood flow and lymph flow [7]. Moreover, similar to ICG, near-infrared fluorescent resin also emits fluorescence, and clinical applications of new devices using this agent are increasing [8]. Fluorescent clips with built-in near-infrared fluorescent resin that emit fluorescence have been used in laparoscopic surgery for colorectal cancer and stomach cancer, and their usefulness has been reported [8], [9]. Clips placed in the stomach and large intestine are visualized via fluorescence through the intestinal canal.

Similar to fluorescent clips, the NIRC used in this case also emits fluorescence when irradiated with near-infrared light, thus facilitating the visualization of the ureter.

To prevent ureteral injury when severe inflammation, such as that observed in diverticular disease, is expected, preoperative ureteral catheter placement is commonly performed, but the effectiveness of this measure has not been established [4]. No evidence of a ureteral injury prevention effect of ureteral stent placement has been reported in the field of gynecology [10]. Particularly in laparoscopic surgery, surgeons cannot rely on the sense of touch. Therefore, conventional ureteral stents cannot be identified manually and are less effective for ureteral navigation.

After the ureter reaches the bladder, it runs through the muscle layer until it opens into the bladder. The tissue surrounding the ureteral orifice was thick, which complicated the fluorescence emission, but the position of the ureteral orifice could be confirmed by pressing the bladder wall with forceps.

One report of sigmoidovesical fistula treatment using NIRC have been published. The course of the ureter was easily and quickly identified by the green fluorescence from the ureteral catheter during laparoscopic surgery for fistulas associated with diverticulitis, where severe inflammation and dense fibrosis were present [11]. This device can be considered extremely useful in the treatment of this disease, which carries a high risk of ureteral injury.

## 4 Conclusion

The laparoscopic sigmoidectomy could be safely performed by visualizing the pathway of the ureter with the fluorescence produced by near-infrared light. We report this method here because it could be considered especially useful.

## Funding statement

There were no sources of funding for this work.

## Ethical statement

This study is a case report and does not necessarily require ethical review. However, fluorescence navigation surgery was approved (approval No. 2020-3) by the Research Ethics Committee of the Kawaguchi Municipal Medical Center, Saitama, Japan.

## Informed consent

Written informed consent was obtained from the patient for the publication of this case.

## Guarantor

Atsuko Okamoto and Ryu Shunjin are guarantor.

## Provenance and peer review

Not commissioned, externally peer-reviewed.

## CRediT authorship contribution statement

Atsuko Okamoto authored the manuscript.

Shunjin Ryu, Atsuko Okamoto, Keigo Hara, Sho Ohno, Yusuke Sasaki and Taketo Ichinose (Department of Urology, Kawaguchi Municipal Medical Center) performed the surgical procedures.

Ms. Keika Iijima, for her assistance with the colorectal database.

## Declaration of competing interest

The authors declares that there is no conflict of interest regarding the publication of this paper.

## References

[1] Marney L.A., Ho Y.H. (2013). Laparoscopic management of diverticular colovesical fistula: experience in 15 cases and review of the literature. Int. Surg..

[2] Collins D., Winter D.C. (2008). Elective resection for diverticular disease: an evidence-based review. World J. Surg..

[3] Tomizawa K., Toda S., Tate T. (2019). Laparoscopic surgery for colovesical fistula associated with sigmoid colon diverticulitis: a review of 39 cases. J. Anus Rectum Colon.

[4] Chiu A.S., Jean R.A., Gorecka J., Davis K.A., Pei K.Y. (2018). Trends of ureteral stent usage in surgery for diverticulitis. J. Surg. Res..

[5] Matsumura M., Takahashi K., Funayama Y., Saijo F., Ikezawa F. (2013). A case of colon diverticulitis with concomitant sigmoidovesical fistulae extending to the bladder trigone. Jpn. Soc. Coloproctol..

[6] (2019). A case of partial cystectomy using laparoscopy endoscopy cooperative surgery (LECS) to treat a vesicosigmoidal fistula. The Japan Society of Coloproctology.

[7] Schaafsma B.E., Mieog J.S., Hutteman M. (2011). The clinical use of indocyanine green as a near-infrared fluorescent contrast agent for image-guided oncologic surgery. J. Surg. Oncol..

[8] Anayama T., Sato T., Hirohashi K. (2020). Near-infrared fluorescent solid material for visualizing indwelling devices implanted for medical use. Surg. Endosc..

[9] Narihiro S., Yoshida M., Ohdaira H. (2020). Effectiveness and safety of tumor site marking with near-infrared fluorescent clips in colorectal laparoscopic surgery: a case series study. Int. J. Surg..

[10] Chou M.T., Wang C.J., Lien R.C. (2009). Prophylactic ureteral catheterization in gynecologic surgery: a 12-year randomized trial in a community hospital. Int. Urogynecol. J. Pelvic Floor Dysfunct..

[11] Osumi W., Yamamoto M., Taniguchi K., Masubuchi S., Hamamoto H., Ishi M., Izuhara K., Tanaka K., Okuda J., Uchiyama K. (2021 May 28). “Clinical experience with near-infrared ray catheter, a fluorescent ureteral catheter, on laparoscopic surgery for colon diverticulitis”: a case report. Medicine (Baltimore).

